# Nanosecond timing and synchronization scheme for holographic pump–probe studies at the MID instrument at European XFEL

**DOI:** 10.1107/S1600577521003052

**Published:** 2021-04-20

**Authors:** Markus Osterhoff, Malte Vassholz, Hannes Paul Hoeppe, Juan Manuel Rosselló, Robert Mettin, Johannes Hagemann, Johannes Möller, Jörg Hallmann, Markus Scholz, Robert Schaffer, Ulrike Boesenberg, Chan Kim, Alexey Zozulya, Wei Lu, Roman Shayduk, Anders Madsen, Tim Salditt

**Affiliations:** a Institute for X-ray Physics, University of Göttingen, Friedrich-Hund-Platz 1, 37077 Göttingen, Germany; b Third Institute of Physics, University of Göttingen, Friedrich-Hund-Platz 1, 37077 Göttingen, Germany; c Deutsches Elektronen Synchrotron – DESY, Notkestrasse 85, 22607 Hamburg, Germany; d European X-ray Free-Electron Laser Facility, Holzkoppel 4, 22869 Schenefeld, Germany

**Keywords:** time-resolved holographic imaging, European XFEL

## Abstract

Single-pulse holographic imaging at XFEL sources with 10^12^ photons delivered in pulses shorter than 100 fs reveal new quantitative insights into fast phenomena. Here, a timing and synchronization scheme for stroboscopic imaging and quantitative analysis of fast phenomena on time scales (sub-ns) and length-scales (≲100 nm) inaccessible by visible light is reported. A fully electronic delay-and-trigger system has been implemented at the MID station at the European XFEL, and applied to the study of emerging laser-driven cavitation bubbles in water. Synchronization and timing precision have been characterized to be better than 1 ns.

## Abstract 2

A timing scheme for single-pulse holographic pump–probe imaging at the MID instrument at the European XFEL is presented and characterized.

## 1. Introduction   

Strongly driven cavitation bubbles in liquids exhibit a range of interesting non-linear effects, from optical breakdown and the emission of a shockwave to violent collapse and sonoluminescence (Vogel *et al.*, 1996*a* ▸; Gaitan *et al.*, 2010 ▸). For well controlled experiments, laser-driven cavitation events are analysed using optical and acoustical means (Vogel *et al.*, 1996*b* ▸; Johansen *et al.*, 2017 ▸). The dynamics of laser-driven plasma, expanding and collapsing vapour bubbles have been studied by, for example, optical high-speed cameras with up to 100 million frames per second (Lindau & Lauterborn, 2000 ▸). However, physical parameters of plasma and vapour in the early states are difficult to quantify by optical means, both due to limited temporal-spatial resolution and because optical imaging cannot directly access mass densities or gas pressures. For example, the time evolution of the bubble radius *R*(*t*) close to the collapse time is yet unknown, but certainly smaller than 100 nm.

These problems can be overcome, however, by single-pulse holography with intense X-ray light pulses (Hagemann *et al.*, 2021 ▸). X-ray free-electron lasers (XFELs) (Altarelli *et al.*, 2007 ▸; Saldin *et al.*, 2000 ▸) allow an instantaneous hologram to be acquired using exposure times below 100 fs. Thanks to small detector pixel sizes below 10 µm and a magnifying imaging system, a spatial resolution below 100 nm is achievable. Last, but not least, the reconstructed phase information directly quantifies the (projected) electron density or mass density (Gabor, 1948 ▸; Cloetens *et al.*, 1996 ▸), even for organic matter (Giewekemeyer *et al.*, 2011 ▸). Single-pulse holograms offer a route to study ultra-fast processes with time-scales down to picoseconds and below. When carried out in a stroboscopic pump–probe scheme, repetitive holographic imaging delivers a time-series of the evolution of relevant physical quantities such as the radial density profile ρ(*r*, τ). A detailed analysis of the performed experiments is reported elsewhere (Vassholz *et al.*, 2021 ▸).

**Figure 1:**
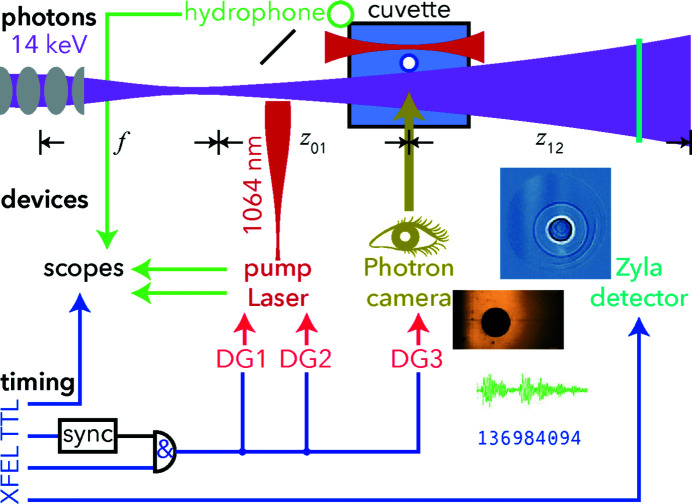
Overview sketch of the laser-driven cavitation X-ray imaging experiments carried out at the MID station of the European XFEL. The 1064 nm pump laser pulses excite cavitation events in purified water inside the cuvette. The electron density profiles of the expanding bubbles are probed by X-ray holography with well defined and adjustable time delays of a few ns up to several µs; at lower time-resolution, optical microscopy images are recorded. Using a microphone, the bubble lifetime is measured.

Here we describe and analyse an electronic timing scheme with resolution better than 1 ns for pump–probe studies on laser-driven cavitation in water. The time-resolution is limited by the laser pulse duration of 6 ns. Hardware to achieve synchronization and control the delay had been chosen and their compatibility with the required delay and jitter goals was tested. The total jitter is determined to be less than 0.4 ns; this value is, however, an upper bound given by the hardware used. An overview sketch of the experiment can be found in Fig. 1 ▸.

This paper is organized as follows. First, we briefly review the European XFEL facility and the MID end-station; then we describe the performed experiments, the optical scheme, and the detectors. In Section 2.4, the delay and synchronization chain is described, and results on performance characterization are shown in Section 3. Before the paper concludes with a summary and outlook, energy fluctuations of the laser-driven cavitation bubbles are analysed.

## 2. Experimental setup   

### 2.1. European XFEL MID end-station   

Experiments were carried out at the Materials Imaging and Dynamics (MID) instrument of the European X-ray Free-Electron Laser (EuXFEL) in Schenefeld, Germany. The European XFEL is a hard X-ray free-electron laser developed for high-repetition-rate experiments delivering up to 27000 pulses per second (Altarelli *et al.*, 2007 ▸).

This design value of 2700 pulses per train at a pulse-separation down to 222 ns and train-repetition rate of 10 Hz has recently been achieved. These trains repeat with a rate of 10 Hz, with 99.4 ms dead-time, *e.g.* for detector read-out and re-charging of the experimental systems. For the experiments discussed here, however, only a single or two pulses per 10 Hz train were used.

In the European XFEL linear accelerator, multi-GeV electrons are directed into insertion devices (undulators) with a magnetic length of up to 175 m, where self-amplified spontaneous emission (SASE) radiation pulses of high spatial and temporal coherence are generated (Saldin *et al.*, 2000 ▸). In addition, the X-ray pulses are very short in time (≲100 fs), thus allowing for pump–probe experiments of very high temporal resolution. The photon flux per pulse is comparable with synchrotron radiation fluxes per second; this allows for single-pulse holographic imaging experiments to capture the time-evolution of the stroboscopically probed specimens with real-space images (Gabor, 1948 ▸; Cloetens *et al.*, 1996 ▸). From reconstructed holograms, phase-shifting structures next to absorbing ones also become visible. Single-pulse holography thus becomes an enabling technique to study fast processes in optically thin samples, *e.g.* biological matter (Giewekemeyer *et al.*, 2011 ▸).

The MID instrument is situated in the SASE-2 section of the EuXFEL and designed for experiments between 5 keV and 25 keV (Madsen *et al.*, 2013 ▸, 2021 ▸). For the experiments p2207 (14 keV) and p2544 (17.8 keV), the machine was tuned for the so-far highest photon energies. The X-ray pulse energy was measured to be fluctuating around 610 ± 260 µJ.

The CRL-1 system is the first component of the SASE-2 beamline and can be used for pre-focusing or collimating the beam. Spot sizes below 200 µm in the sample plane can be achieved. A microfocused beam can be obtained with the CRL-2 system (Madsen *et al.*, 2013 ▸, 2021 ▸). The sample chamber can be evacuated; here, instead, experiments were carried out in air.

To achieve a large magnification for high-resolution holograms, a stack of nano-CRLs including an aberration correcting phase plate (Seiboth *et al.*, 2017 ▸) was placed in front of the sample stage. The imaging detector was placed about 9.7 m downstream from the sample; the flight path between sample and detector was evacuated to avoid background from air scattering. For optical pumping of cavitation events, an infrared laser system was installed close to the sample; see below for details.

For more information refer to Table 1 ▸.

### 2.2. Excitation laser, cavitation events   

To stimulate cavitation events, a nanosecond infrared laser system was used. More specifically, the 1064 nm emission from a Litron Lasers Nano L 200-10 (Litron Lasers, Rugby, UK) was focused into the purified water inside the cuvette. The pulse duration was about 6 ns. For beam transport, standard optical components were used, including a telescope to widen the beam in front of the focusing optic. For focusing, a singlet lens was used while a numerical aperture of about 0.2 is reached in both experiments. The focused laser spot size is expected to exceed the Gaussian limit of 2 µm (FWHM), since the focusing geometry is slightly affected during fine adjustment of the X-ray and laser alignment. In addition the converging beam was reflected by a drilled-through flat mirror which is expected to slightly distort the beam.

The laser intensity was set to about 21%–25%, corresponding to about 17 mJ to 24 mJ per pulse. The seeding rate of cavitation events is about 23% with an RMS variation of 3%; multi-bubble excitation is observed for 30% of the cavitation events.

Typical cavitation bubbles observed have a maximum radius of about 500–750 µm and lifetimes of 100–150 µs; see Section 3.2 for a detailed analysis.

### 2.3. X-ray focusing and detection   

#### 2.3.1. Compound refractive lenses   

For focusing of the X-rays, a stack of 50 compound refractive lenses (CRLs; made from beryllium with an apex radius of *r* = 50 µm and a geometrical aperture of *D* = 300 µm) with a focal length of *f* = 298 mm was used. The focal length is measured from the centre of the CRL stack. The CRL stack is aberration corrected by a phase-plate (Seiboth *et al.*, 2017 ▸), thus yielding a diffraction-limited focus size of 78 nm (p2207) and 94 nm (p2544) (Hagemann *et al.*, 2021 ▸); for further details, see Table 1 ▸.

#### 2.3.2. X-ray detectors   

For detection of holograms, a scintillator-based sCMOS camera (Andor Zyla 5.5, Oxford Instruments, Abingdon, United Kingdom) with 5 Mpixels of 6.5 µm pitch was placed about 9.7 m downstream of the cuvette. This leads to a geometrical magnification of 62 and, accordingly, to a virtual pixel size of 106 nm. The Fresnel number of the wave-optical imaging system is about *F* ≃ 7.6 × 10^−4^. See Table 1 ▸ for a summary of these and additional parameters for both experiments. The given corrected Fresnel number is based on visual inspection of different reconstructions and includes corrections for, for example, a curvature of the incoming wavefront by upstream optics, uncertainties in the measured distances, and possible distortions by the visual light optics of the X-ray detector.

**Figure 2:**
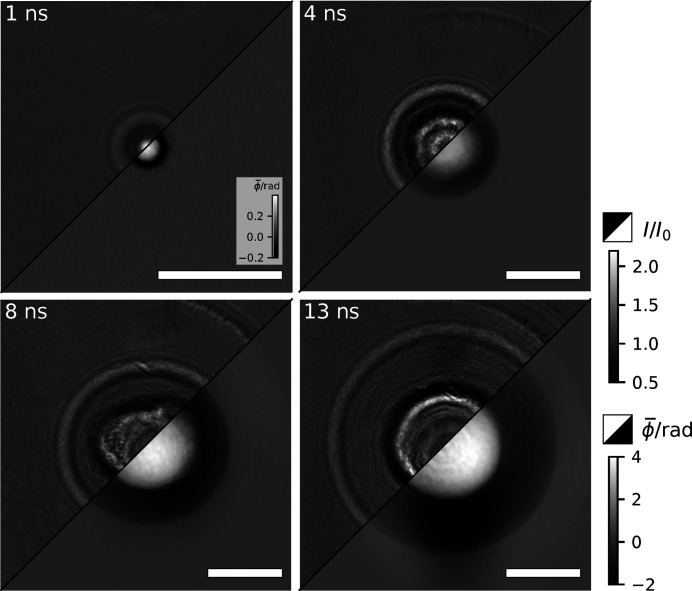
Flat-field corrected holograms (upper left triangles) and reconstructed phase (lower right triangles) of four cavitation events at increasing time delay. The scale bars denote 25 µm.

Exemplary flat-field corrected holograms and phase reconstructions of four cavitation events with increasing time delay are shown in Fig. 2 ▸. For further details, the reader is referred to Hagemann *et al.* (2021 ▸).

#### 2.3.3. Optical camera and microphone   

In addition to the X-ray detector, a high-speed optical camera (Photron Fastcam SA5, up to 50000 frames per second at 512 × 272 pixels; Photron, Tokyo, Japan) was also used to obtain image series of the bubble growth and collapse processes. Based on the images, the maximum bubble radius can be measured to estimate the deposited energy per individual cavitation event.

As an event marker, a piezo-electric microphone was attached to the water cuvette, serving as a microphone and recording acoustics generated by the plasma ignition and the bubble collapse. From these, the bubble lifetime τ ≃ 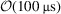 can be extracted. Correlating τ to the maximum bubble radius obtained from the Photron camera images allows estimating the deposited energy, and distinguishing differently seeded bubbles. The reader is referred to Section 3 below and Vassholz *et al.* (2021 ▸) for more details.

### 2.4. Time delays and triggering   

**Figure 3:**
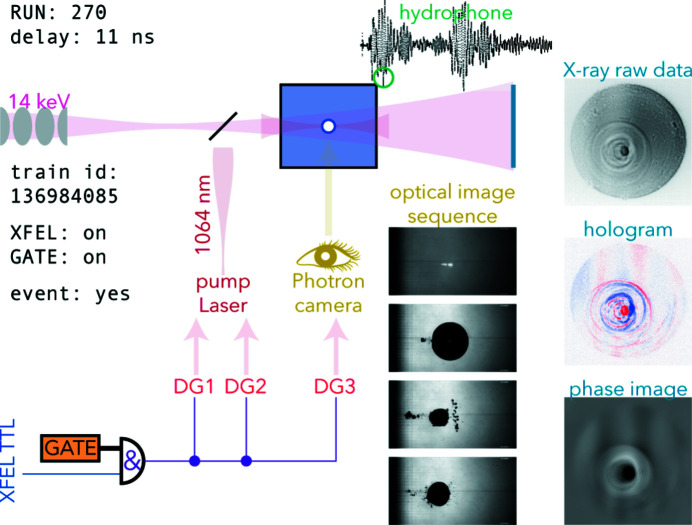
One particular cavitation event (the full animation is available as an animated movie in the supporting information), shown as optical microscopy (central column of grey-scale images) and X-ray detector images (top image, right), including normalized hologram (blue–red coloured image) and phase image (grey scale, bottom image, right). The cavitation event was detected using the microphone signal (oscilloscope trace, top).

The timing scheme with all trigger signals, the laser pump pulses, the X-ray probe flashes, and detector image data including phase reconstructions, is available as an animated movie in the supporting information. One still image is shown in Fig. 3 ▸; here, the AND gate is switched on, an actual cavitation event was pumped and recognized by the microphone, and corresponding data taken with optical camera and X-ray detector are shown. Also, the normalized hologram and the reconstructed phase image are shown.

#### 2.4.1. Cabling scheme   

With the timing specifications of the European XFEL facility in mind, the time-resolution of <1 ns in this work is very modest; also the requirements on cables and connections are rather low. Hence, usual co-axial RG 58 C/U cables with 50 Ω impedance and BNC connections have been used. At a length of 20 m, they are rated for a bandwidth of ∼340 MHz (at 3 dB amplitude attenuation); this allows for nearly unaffected delivery of the signals to oscilloscopes. Inside the control hutch, cables are much shorter and the bandwidth is of virtually no concern.

**Figure 4:**
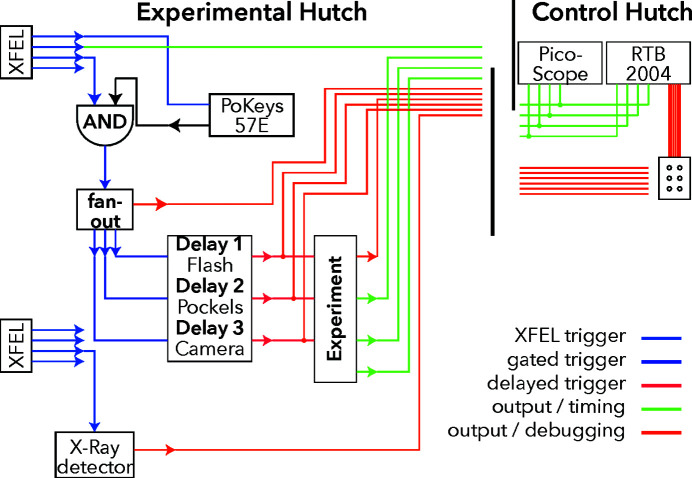
Schematics of the BNC cabling; for details see the text.

The full cabling scheme is sketched in Fig. 4 ▸. Blue lines carry the initial EuXFEL trigger signals (see also the top part of Table 2 ▸), continuously at 10 Hz. The purple lines show the gated signals, which are only active during measurement. Lines shown in red are electronically delayed to define the current time delay. The green lines are used to measure and quantify the timing using the PicoScope; lines depicted in orange carry debugging signals to the oscilloscope for visual inspection (see below for details on the oscilloscopes).

#### 2.4.2. Fan-out and AND gate   

To synchronize all equipment, the *same first pulse* has to be propagated deterministically to all devices. To this end, a PoKeys57E board was used to ‘measure’ the 10 Hz period of the EuXFEL, and an AND gate (74ACT08 with AD8009 amplifier) was introduced before a 1:4 TTL fan-out (PRL-414B, Pulse Research Lab, Torrance, California, USA). For the fan-out, the bandwidth of >80 MHz, a (typical) rise time of 2.2 ns and a channel skew of 0.5 ns, the timing requirements of the experiment are easily met. For the AND gate and amplifier, a propagation delay of 3.55 ns and a rising time of 2.3 ns was measured. The combined delay of fan-out and AND gate was measured to be 19.0 ns with RMS 0.1 ns.

#### 2.4.3. Delay generators   

A pair of low jitter delay generators DG535 (Stanford Research Systems) was utilized to define precisely timed pulses for the laser flash lamp (*T*_flash_), the Pockels cells (*T*_Pockels_), and the high-speed optical camera (*T*_photron_). The delay between *T*_flash_ and *T*_Pockels_ was kept constant at 160 µs to maximize the lasing power. Then, by shifting *T*_flash_ with respect to EuXFEL’s trigger *T*_0_, the delay between pumping laser pulse and probing X-ray pulse was varied from negative values (first X-ray, then laser) to positive values, with a dense sampling close to 0 ns and a coarse sampling up to 11 µs. This is possible since the actual EuXFEL photon train arrives after the electronic trigger signal.

The time delay XFEL trigger 4 of the X-ray detector was preset to the value 5212395 FPGA clock cycles; the exposure time was set to half the EuXFEL period, *i.e.* 50 ms. Since the EuXFEL pulses have a duration on the order of 100 fs and repeat with 10 Hz, the actual exposure length and phase are non-critical for the experiment. It has to be noted, though, that the event stamping with respect to the trainId needs to be checked. In the presented experiments, the X-ray detector was lagging behind by one trainId, but this has been taken care of in the data analysis.

#### 2.4.4. Oscilloscopes   

All timing signals were visually inspected using a stand-alone oscilloscope RTB 2004 (Rohde & Schwarz, Munich, Germany; four analog channels plus six out of 16 digital on–off-channels with a bandwidth of 300 MHz; sampling rate up to 2.5 GS s^−1^); for data processing, the four analog channels were also digitized by a USB oscilloscope (PicoScope 6402C, Pico Technology, St Neots, UK: four channels, bandwidth 250 MHz, per-channel sampling rate up to 1.25 GS s^−1^). The four analog channels are:

A – EuXFEL trigger pulse (*T*_0_, XFEL trigger 1);

B – Laser Pockels out (*T*_Pockels_);

C – Photron out (*T*_photron_);

D – Microphone signal (event marker).

On channel D, residual noise signals emerging from the Pockels cells can also be seen.

The six digital channels are mainly used for debugging/visual inspection of input signals or uncritical signals (*i.e.* laser flash in/output and X-ray detector output).

Apart from timing and event marking, the microphone signal is also used to quantify the deposited energy, and, derived from that, the maximum bubble size. The data have therefore been calibrated to Photron images; the camera was not used during all runs because of its slow data transfer.

#### 2.4.5. Triggers from EuXFEL   

**Figure 5:**
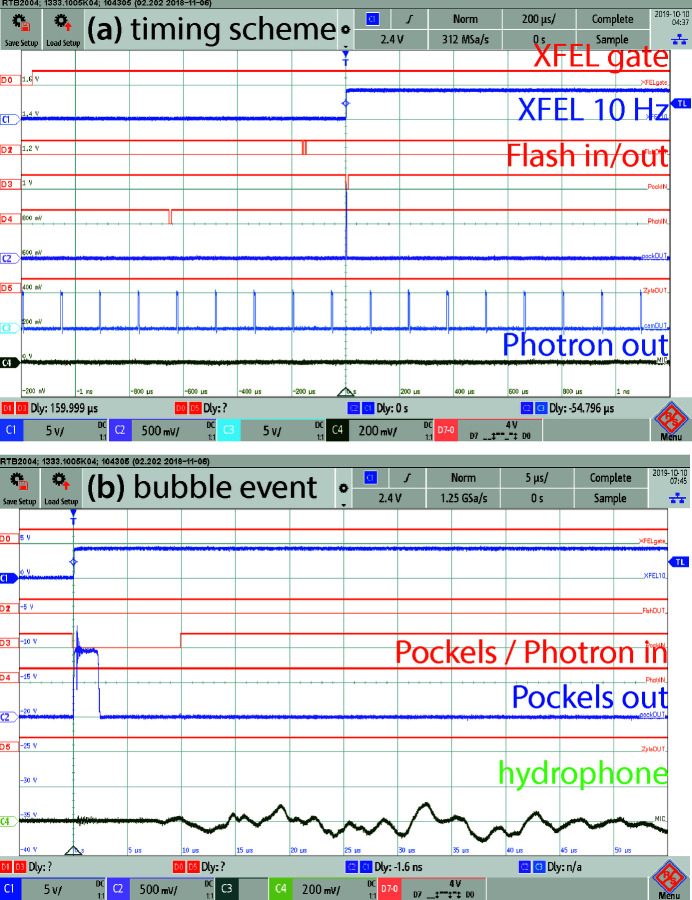
Oscilloscope traces: analog channels are shown in individual colours, digital channels in orange. (*a*) The overall timing scheme shows the master EuXFEL trigger (second trace) and the trigger pulses to/from laser (flash and Pockels), optical Photron and X-ray Andor Zyla camera. (*b*) A bubble event is registered by the microphone (last trace). Residual noise from the Pockels cells (sixth trace) has been used to measure actual time delays and jitter (see also Fig. 6 ▸).

The trigger signal XFEL trigger 1 (*T*_0_) from the EuXFEL was recorded along with output signals from laser Pockels (*T*_Pockels_) and the Photron camera (*T*_photron_) and the microphone to measure per-shot delay values of the experiment. A signal before the AND gate and fan-out had been chosen as a reference to catch jitter of this equipment as well. For the synchronization, the signal XFEL trigger 3 had been monitored before the start of a run in order to switch the AND gate at a definite phase. Then signals from XFEL trigger 2 could pass the AND gate and fan-out, to be propagated to three delay generator channels for the actual experiment. See Table 2 ▸ for more details, Fig. 4 ▸ for a schematic of the cabling, and Fig. 5 ▸ for exemplary oscilloscope traces.

The EuXFEL trigger signals are generated by local FPGA boards, which are interconnected via glass fibre to the master oscillator at DESY. They allow for per-output shifts in clocks of 9.23 ns.

#### 2.4.6. Spatial and temporal overlap   

To find the temporal overlap, a photodiode AXUVHS11 (Opto Diode, Camarillo, California, USA; rise time 700 ps) was placed into the fully diverged laser and EuXFEL beam at ∼8 m distance from the interaction region. Using the DG535 delay generators, the pump-laser was shifted to temporally overlap with the EuXFEL flashes. The correct timing was later confirmed during the pump–probe experiments.

To spatially align the pumping laser beam and probing EuXFEL beam, a motorized tungsten (W) needle was placed close to the interaction region and then aligned in X-ray absorption contrast. Then the optical laser was aligned onto the needle using motorized mirrors. For the liquid jet experiments, the thin water jet (diameter around 30 µm) was aligned with respect to the needle, too. The correct spatial alignment was confirmed and corrected during the actual pump–probe experiments.

## 3. Characterization and control   

### 3.1. Jitter and delay measurements   

The jitter of the synchronization chain (AND gate, fan-out) and laser have been determined from characteristic noise induced by the Pockels cells into a BNC cable. The PicoScope was triggered by the EuXFEL *T*_0_, and the time delay to the Pockels signal has been quantified for a particular delay campaign. The PicoScope was operated at a sampling interval of 0.8 ns and with an analog bandwidth of 250 MHz; the estimated jitter of 0.4 ns is thus an upper bound. The nominal and measured delay values are in agreement within a margin of 0.2 ns for analog channels and within 0.5 ns for digital channels.

Also, delays and jitter introduced by the timing equipment and cables have been measured using the RTB scope at a sampling rate of 0.8 ns down to 0.4 ns (depending on settings; the analog bandwidth is 300 MHz). The channel skew of the TTL fan-out including two long BNC cables was measured to be 0.6 ns. BNC extensions to reach the optical high-speed Photron camera (+24 ns) and the microphone at the cuvette (+26 ns) have been measured, as well as the ‘0’-delay of the DG535 delay generators (88.4 ns to 90.5 ns, depending on channel and device). In addition to the four analog channels, the RTB offers a logic probe with up to 16 channels; the propagation delay (probe plus ribbon cable) was determined to be 7.7 ns. From the measurements, all delay values given in this paragraph can be considered to be ‘constant’ within 0.2 ns (standard deviation of the RTB’s delay measurements).

**Figure 6:**
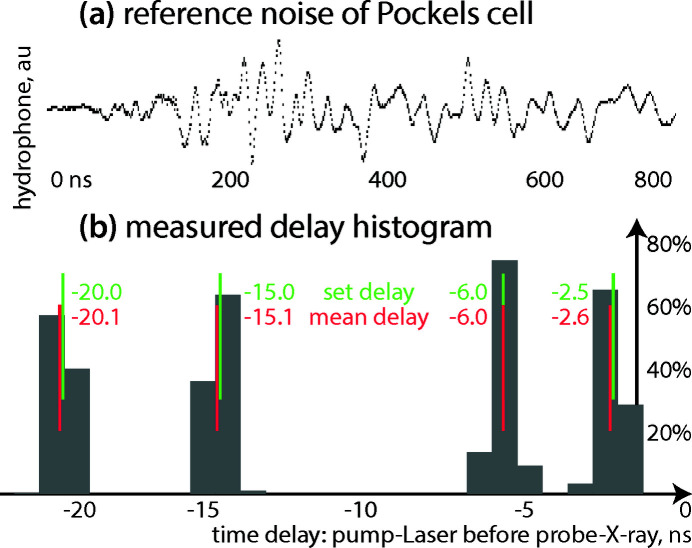
Pockels cell jitter measurement via noise correlation. (*a*) The Pockels effect induces a strong noise-like, but reproducible, pattern in a long BNC cable observed with the oscilloscopes. The reference signal shown here is correlated in the time-domain to individual signals from the time delay runs. The scope was triggered using the primary EuXFEL trigger *T*_0_. (*b*) Corresponding delay histograms (grey bars) for certain nominal time delays (green lines) and average delays from cross-correlations (red lines). All measurements agree within the sampling interval of 0.8 ns. It can thus be concluded that the jitter between primary EuXFEL trigger *T*_0_ and the actual *T*_Pockels_ is σ ≃ 0.4 ns.

Apart from these individual and *ex situ* measurements, the full delay jitter can also be quantified. For that we make use of the strong noise-like, but reproducible, pattern obtained in long BNC cables close to a laser operated with Pockels cells. Fig. 6 ▸(*a*) shows a reference trace of the signal. This has been correlated in the time-domain to measurements at different nominal delay values to directly measure the actual delay and its jitter. Histograms of representative delays are shown in Fig. 6 ▸(*b*), with a bin width of 0.8 ns corresponding the Pico­Scope’s sampling rate. The calculated mean values are within ±0.3 ns of the nominal values; the histogram distributions show a width of ∼0.4 ns. We conclude that the jitter between the primary EuXFEL trigger *T*_0_ and the actual *T*_Pockels_ is below the time resolution goal of 1 ns. The PicoScope was triggering on the EuXFEL reference before the AND gate, so this measurement includes the full delay chain comprising AND gate, fan-out, DG535, and the laser itself.

### 3.2. Energy fluctuation of bubble seeding   

Holographic images and reconstructed phases of single cavitation events at a single time delay are pictorial; to study the time evolution, a series of images along a certain time delay range is needed. But the quantitative analysis of such stroboscopic experiments relies on the assumption of repetitive events. For laser seeded bubbles, the time point is well known (see analysis above) and the pulse energy can be estimated to be rather constant. But the deposited energy fluctuates per shot; hence cavitation bubbles of different sizes and hence time evolutions are produced within a single time delay run.

**Figure 7:**
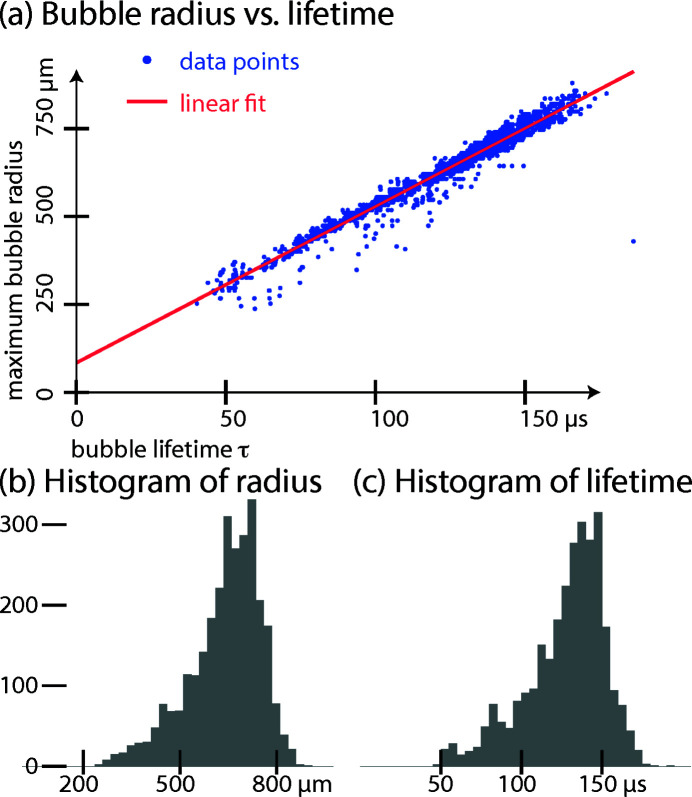
Energy fluctuations of cavitation events: (*a*) maximum bubble radius *R*_max_ as a function of bubble lifetime τ. (*b*, *c*) Histograms of the bubbles’ maximum radius and lifetime distributions, respectively.

Such a family of bubbles can be described by a single parameter, *i.e.* the deposited energy *E* (Vogel *et al.*, 1996*a* ▸). The energy is not measured directly, but quantified from the recorded maximum radius *R*_max_, and with the microphone data the bubble lifetime τ (time duration between plasma ignition and collapse sound) is available. From model calculations and measurements it is known that *E*, *R*_max_, and τ are bijective functions of one another (at least within reasonable limits). Although the actual *E*(*R*_min_) is unknown here, we can distinguish and sort different bubbles by their lifetime. Maximum radius *R*_max_ and bubble lifetime can be related via 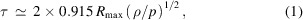
with density ρ and pressure *p*, according to the rather simple Rayleigh model (Rayleigh, 1917 ▸). Note that we assume a symmetry between growth and collapse, which yields a factor of 2. While more advanced models include, for example, local pressure variations, the linear relations hold (Plesset & Prosperetti, 1977 ▸). Fig. 7 ▸(*a*) shows a scatter plot of maximum radius *R*_max_ (taken from the Photron camera image series) and the lifetime τ (extracted from the microphone traces). Since only a part of the data streams include Photron images due to slow read-out, we have used the linear fit model,
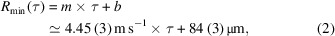
to extrapolate and tag the events with an ‘energy’ label. The offset *R*_0_ ≃ 84 µm can only in part (about 30 µm to 80 µm, depending on threshold values) be attributed to the initial plasma size of the cavitation seeding event. Figs. 7 ▸(*b*) and 7(*c*) show histograms of the bubbles’ maximum radius and their lifetime, respectively.

## 4. Summary and outlook   

Single-pulse holographic X-ray imaging has become a reality thanks to the extreme brilliance of XFEL sources. We have designed and analysed an electronic delay scheme for time-resolved pump–probe experiments at a time-resolution of better than 1 ns. The delay chain is fully electronic, providing more flexible and easy manipulation during the experiment, compared with optical delay lines. The overall jitter between incoming EuXFEL trigger pulse and secondary electronic signal generated by the laser’s Pockels cell has been measured *in situ* and quantified to be better than 0.4 ns (RMS); this estimate is an upper bound due to the oscilloscope’s limited sampling rate.

As an event marker, a microphone signal has been recorded per X-ray pulse; these data are used both to find cavitation events and to quantify the deposited energy from the bubble lifetime. The lifetime correlates well to the bubbles’ maximum radii, which are another observable related to energy. The deposited energy is needed in the analysis to group cavitation events into ‘energy bins’.

For moderate time resolution using, for example, ns-laser systems, the presented scheme is sufficient. A fs-laser system equipped with optical delay lines is installed and commissioned at the European XFEL to access even shorter time scales.

The algorithm for single-pulse phase reconstruction of holograms with fluctuating intensity profiles from pulse to pulse can be found in Hagemann *et al.* (2021 ▸). Data taken at European XFEL are available – Hagemann *et al.* (2019*a* ▸,*b* ▸).

## Supplementary Material

AVI video (Full animation of the still-image shown in figure 3 illustrating the timing scheme.). DOI: 10.1107/S1600577521003052/ve5135sup1.avi

## Figures and Tables

**Table 1:** Physical and geometrical parameters of the holographic pump–probe studies

	p2207	p2544	
Parameter	May 2019	October 2019	Unit
SASE 2	25	31	Undulator cells
Electron energy	14.0	16.5	GeV
Photon energy	14.0	17.8	keV
Pulse energy	∼610	≤700	µJ
Repetition rate	10	10	Hz (single bunch)

CRL focal length	298	475	mm
CRL numerical aperture	0.42	0.27	× 10^−3^
Distance: focus–sample	146.5	276.8	mm
Distance: sample–detector	8858	9665	mm
Demagnified pixel size	97	181	nm
Fresnel number *F* ^1^	7.6	17.5	× 10^−4^
WAXS detector distances	–	∼300	mm
WAXS detector pixel size	–	50	µm

Laser wavelength	1064	1064	nm
Laser pulse energy	24	17	mJ
Laser spot size	2	2	µm (FWHM)
Laser pulse duration	6	6	ns

**Table 2:** Triggers from EuXFEL and (delayed) triggers to the experiment

Name	Scope	Purpose
XFEL trigger 1	A	*T*_0_ reference trigger for oscilloscopes
XFEL trigger 2		Input to delay chain (below)
XFEL trigger 3		Trigger for AND gate synchronization
XFEL trigger 4		Trigger for X-ray detector (Zyla)

AND gate, then		
fan-out-0	D0	Gated trigger pulses
fan-out-1		To DG1
fan-out-2		To DG2
fan-out-3		To DG3

DG1	D1	Input laser flash
	D2	Output laser flash
DG2	D3	Input laser Pockels
	B	Output laser Pockels
DG3	D4	Input Photron camera
	C	Output Photron camera
	D	Output microphone
	D5	Output X-ray detector (Zyla)
